# Research hotspots and frontiers in non-specific low back pain: a bibliometric analysis

**DOI:** 10.3389/fneur.2024.1464048

**Published:** 2024-10-30

**Authors:** Qiangjian Mao, Yuqing Wang, Shiqi Xu, Desheng Wu, Guomin Huang, Ziru Li, Lin Jiao, Zhenhai Chi

**Affiliations:** ^1^Acupuncture and Moxibustion Department, Affiliated Hospital of Jiangxi University of Chinese Medicine, Nanchang, Jiangxi, China; ^2^Acupuncture and Moxibustion Massage College, Jiangxi University of Chinese Medicine, Nanchang, Jiangxi, China

**Keywords:** non-specific low back pain, bibliometrics, CiteSpace, hotspots, frontiers

## Abstract

**Background:**

Extensive research has been conducted worldwide on non-specific low back pain (NSLBP), some researchers published a bibliometric analysis of NSLBP in 2020, but there have been no supplements or updates since then. Therefore, this study aimed to analyze the research hotspots and frontiers in NSLBP over the last decade.

**Methods:**

Primary sources on NSLBP were obtained from the Web of Science Core Collection database from 2014 to 2023. CiteSpace V6.2. R7 (64-bit) and VOSviewer 1.6.19 software were used to analyze the number and centrality of journals, countries, institutions, authors, references, and keywords, and the functions of co-occurrence and clustering were applied to draw a visual knowledge map.

**Results:**

In the past decade, the annual publication volume of studies on NSLBP has shown an overall upward trend year by year, with obvious temporal stages and great development potential. In total, 2,103 articles contained six types of literature, with the highest proportion being original research articles (1,633 articles, 77.65%), published in 200 journals. *BMC Musculoskeletal Discourses* (90 articles, 4.28%) had the highest number of publications, and the *British Medical Journal* had the highest impact factor (105.7). Furthermore, the United States of America (329 articles, 15.64%) had the highest publication volume, the University of Sydney (139 articles, 6.61%) was the research institution with the highest production, Maher, Chris G (36 articles, 1.71%) was the author with the most published articles, and Hoy, D (571 articles, 27.15%) was the most frequently cited author. The most cited of articles is “Non-specific low back pain” published in the LANCET, with 1,256 citations.

**Conclusion:**

This article summarizes the current research status of NSLBP and predicts future research hotspots and frontiers. In recent years, adolescents have become a high-risk group for NSLBP. Pain neuroscience education, motor control, spinal manipulative therapy, and acupuncture are effective means to treat NSLBP. Biomechanics and trunk muscles as entry points are effective ideas for the treatment of NSLBP pain. Furthermore, anxiety, neck pain, non-specific musculoskeletal pain, fibromyalgia, and musculoskeletal disorders are diseases that are closely related to NSLBP. In the future, attention should be paid to the design of research plans, increasing the research intensity of randomized controlled trials, strengthening follow-up, and the timely updating of guidelines, which will result in higher quality and high-level scientific evidence for research on NSLBP.

## 1 Introduction

According to authoritative statistics, low back pain (LBP) affected approximately 568 million people worldwide in 2019 (1). A systematic review of 56 countries shows that the lifetime prevalence of LBP is as high as 40%; as a result, LBP is one of the most common musculoskeletal disorders (2). In the United States of America (USA), LBP has the highest overall healthcare expenditure, estimated at $134.5 billion in 2016 (£95 billion; €110.5 billion), with a 95% confidence interval (CI) of $122.4 billion to $146.9 billion (3). LBP can be divided into specific and non-specific types. Non-specific low back pain (NSLBP), where the specific pathological and anatomical reasons cannot be determined, accounts for 80–90% of cases (4, 5). In addition, more than 60% of patients may experience pain and disease recurrence after 1 year of onset, progressing to chronic NSLBP. Thus, NSLBP is a major global public health problem that imposes a huge burden on individuals, healthcare, and society, and has been a major cause of disability worldwide for the past 30 years (6–8).

The pathogenesis of NSLBP has not been elucidated. Currently, there are many methods for treating NSLBP in clinical practice, which can be roughly divided into drug treatments and non-drug treatments. Drug therapy is a first-line treatment method for NSLBP and is widely used in clinical practice. Drug therapy usually requires clinical doctors to develop specific medication plans and make necessary adjustments, and commonly includes nonsteroidal anti-inflammatory drugs (NSAIDs), muscle relaxants, and analytical medicine (9–12). However, drug therapy can have certain side effects and uncertain therapeutic effects. For example, NSAIDs can have adverse effects on the cardiovascular and gastrointestinal systems, while analgesic drugs have uncertain long-term efficacy and can become addictive (13). Given the high cost, side effects, and limited efficacy of drug therapy for NSLBP, various NSLBP treatment guidelines and consensus published in recent years recommend the use of non-drug means, including various sports treatments (14–16), physical factor treatments (17), and acupuncture replacement therapy (18, 19).

Bibliometrics is an interdisciplinary science that quantitatively analyzes all knowledge carriers. It uses mathematical and statistical methods to manage literature, analyze the academic level, research direction, and academic trends of different disciplines (20–22).

At present, no bibliometric analysis of NSLBP exists; therefore, this study aimed to explore the hotspots and frontiers of research on NSLBP in the past decade to provide some assistance for future research directions.

## 2 Materials and methods

### 2.1 Source of literature

**Figure 1:**
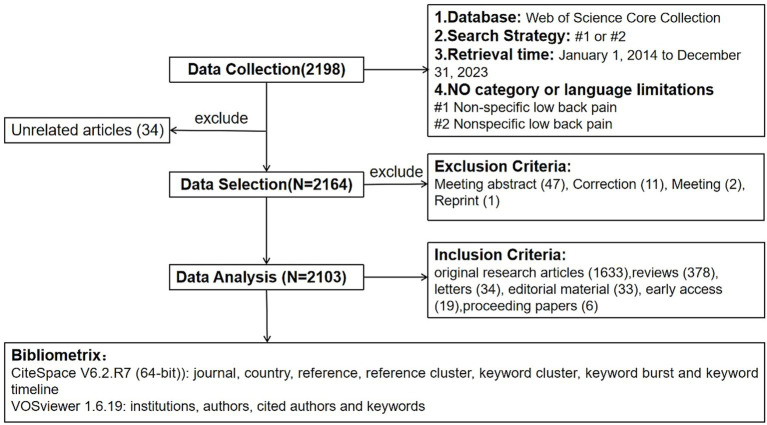
Flow diagram of the included studies.

We performed a bibliometric analysis of research articles on NSLBP published from January 1, 2014, to December 31, 2023, obtained from the Web of Science Core Collection (WOSCC) database, with a search date of March 3, 2024. Our search terms included ST = (Non-specific low back pain) or (Nonspecific low back pain). Searches were not limited to the category or language of literature. Searches were conducted independently by Shiqi Xu and Desheng Wu; when discrepancies arose, they were resolved by Yuqing Wang. A total of 2,198 articles were retrieved, and 34 articles unrelated to the topic were excluded, leaving 2,164 articles. Following a CiteSpace software check, no duplicate articles were detected. To ensure the effectiveness of the research conclusions, short passages or incomplete articles were not included in the analysis. Therefore, we excluded 47 meeting abstracts, 11 corrections, two meetings, and one reprint, resulting in 2,103 articles for the final bibliometric analysis (Figure 1). Data deletion was done as a combination of manual validation and the CiteSpace software. The Web of Science search was sourced from the Jiangxi University of Chinese Medicine Library’s database.

### 2.2 Analysis tools

CiteSpace is a tool used to analyze the scientific literature developed by Dr. ChaoMei Chen in conjunction with the WISE Laboratory. The software is based on co-citation analysis and pathfinding network algorithms to visualize data samples and present the evolution of a specific knowledge domain (23, 24). VOSviewer is a JAVA-based software tool developed by Van Eck and Waltman from the Leiden University Science and Technology Research Center in the Netherlands in 2009. It showcases the knowledge structure and relationships in the research field through co-citation and co-citation principles. Both software tools can visualize the relationships between the literature in the form of a scientific knowledge map. This not only helps us clarify the past research trajectory, research status, and hot topics of a certain field but also reveals the future development direction of the field (25–27). This study used CiteSpace V6.2.R7 (64-bit), VOSviewer 1.6.19, and Excel to determine trends in authors, journals, institutions, countries, and keywords.

## 3 Results and discussion

### 3.1 Analysis of the annual volume of publications

**Figure 2:**
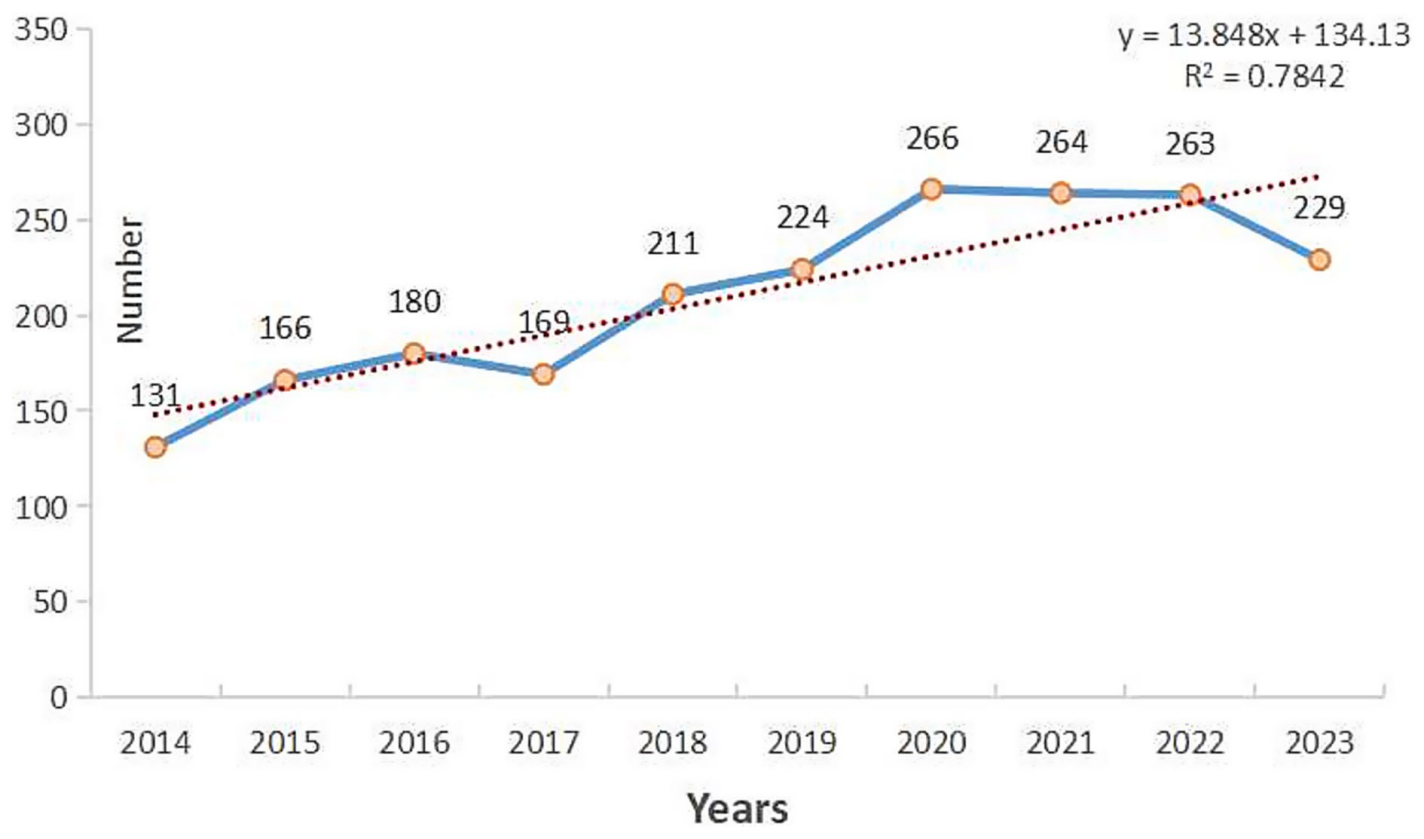
The annual number of publications related to non-specific low back pain (NSLBP) from 2014 to 2023.

The annual number of publications can reflect the time-series change of a scientific issue and is an important indicator in bibliometrics. Excel software was used to produce an annual chart of the distribution of article publications (Figure 2). The number of articles published on NSLBP in the past 10 years has fluctuated slightly; however, overall a steady upward trend was observed, which could be divided into three growth phases. The first phase (2014–2016) was the exploratory period, with the literature increasing from 131 to 180; the second phase (2017–2020) was the accelerated period, with the literature increasing from 169 to 266; and the third phase (2021–2023) was the stabilization period. The factors that lead to these different phases may be the reason for the fluctuation of publication rates in different phases. It is noteworthy that in the second phase, it enters a new height, with an accelerated increase in literature publication, reaching an all-time high in 2020, with 266 annual publications (12.65%). We have demonstrated high reliability of the trend line by calculating the slope (*y* = 13.848*x* + 134.13; *R*^2^ = 0.7842). The research results indicate that NSLBP has received sustained attention from researchers, and the research momentum is strong, with enormous potential.

### 3.2 Analysis of journals and cited journals

The 2,103 articles were categorized into six types of publication. The most common type was original research articles (1,633 articles, 77.65%), followed by reviews (378 articles, 17.97%), letters (34 articles, 1.62%), editorial material (33 articles, 1.57%), early access (19 articles, 0.90%), and proceeding papers (6 articles, 0.29%) (Table 1). The 2,103 articles were published in 200 journals, with the highest number of articles published in BMC Musculoskeletal Disorders (90 articles), followed by the European Spine Journal (68 articles), the Journal of Back and Musculoskeletal Rehabilitation (67 articles), PLoS One (61 articles), and Spine (56 articles); the 6th to 10th positions are shown in Table 2. Referring to the Journal Citation Report 2023 of the American Institute for Scientific Information, we found that among these journals, the journal with the highest impact factor (IF) was the British Medical Journal (IF = 105.7). The most cited of the 2,103 articles is “Non-specific low back pain” published in the LANCET, with 1,256 citations.

**Table 1:** Literature types related to non-specific low back pain (NSLBP).

Rank	Type	Counts (%)	Rank	Type	Counts (%)
1	Original Research Articles	1,633 (77.65)	4	Editorial Material	33 (1.57)
2	Review	378 (17.97)	5	Early Access	19 (0.90)
3	Letter	34 (1.62)	6	Proceedings Paper	6 (0.29)

**Table 2:** Top 10 journals and publications related to non-specific low back pain (NSLBP).

Rank	Publications	Journal	IF (2023)	Rank	Publications	Journal	IF (2023)
1	90	BMC Musculoskeletal Disorders	2.3	6	54	BMJ Open	2.9
2	68	European Spine Journal	2.8	7	48	Musculoskeletal Science and Practice	2.3
3	67	Journal of Back and Musculoskeletal Rehabilitation	1.6	8	39	Medicine	1.6
4	61	PLoS One	3.7	9	39	Physical therapy	3.2
5	56	Spine	3.0	10	38	Trials	2.5

**Figure 3:**
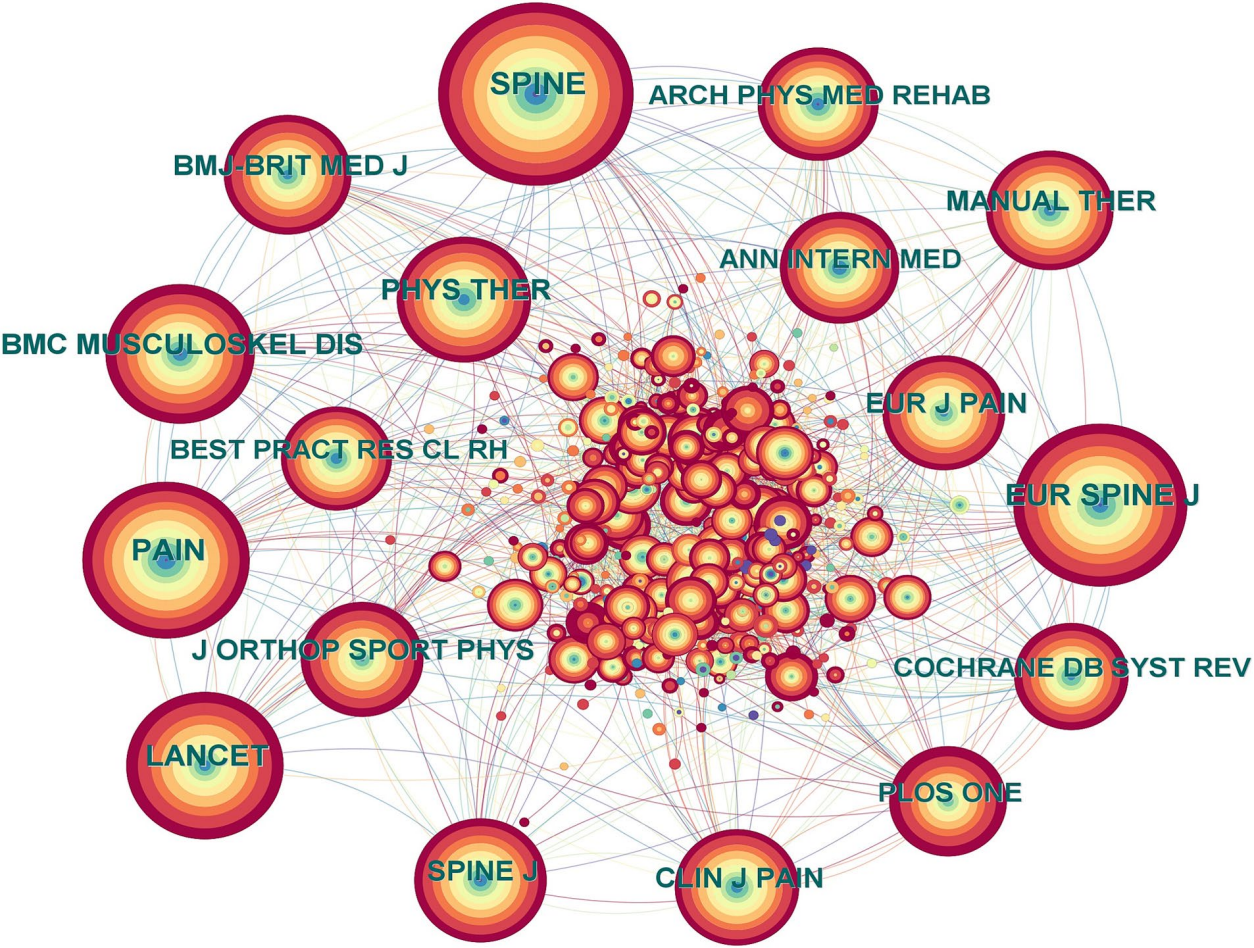
Cited journal map related to non-specific low back pain (NSLBP) from 2014 to 2023.

Combining co-citation and centrality, CiteSpace was used to generate a network map of the cited journals (Figure 3 and Table 3). Centrality is a concept that refers to the degree to which nodes (such as authors) are close to the center in the entire literature network, usually the higher the centrality suggests that the nodes are more important. The nodes in the figure represent the cited journals, and the connections between nodes represent co-cited relationships. The larger the node range, the higher the number of co-citations. The purple ring represents centrality, with nodes with high centrality being important key points. The top journals in terms of frequency and centrality were *Spine* and the *Journal of General Internal Medicine*. To summarize, these two journals had a high citation rate and strong representativeness and authority in this research field, which can provide professional, practical, and evidence-based support for the researchers and practitioners in NSLBP.

**Table 3:** Top 10 cited journals and centrality related to non-specific low back pain (NSLBP).

Rank	Cited Journal	Frequency	Rank	Cited Journal	Centrality
1	Spine	1,692	1	Journal of General Internal Medicine	0.04
2	European Spine Journal	1,362	2	Journal of Neuroscience	0.04
3	Pain	1,275	3	Journal of Alternative and Complementary Medicine	0.03
4	Lancet	1,134	4	Brain	0.03
5	BMC Musculoskeletal Disorders	974	5	Journal of Advanced Nursing	0.03
6	Physical Therapy	860	6	Journal of Epidemiology and Community Health	0.03
7	Spine Journal	791	7	American Journal of Preventive Medicine	0.03
8	BMJ-British Medical Journal	756	8	Clinical Biomechanics	0.02
9	Manual Therapy	738	9	Medicine & Science In Sports & Exercise	0.02
10	Clinical Journal of Pain	735	10	Journal of Physical Therapy Science	0.02

### 3.3 Analysis of countries and institutions

**Figure 4:**
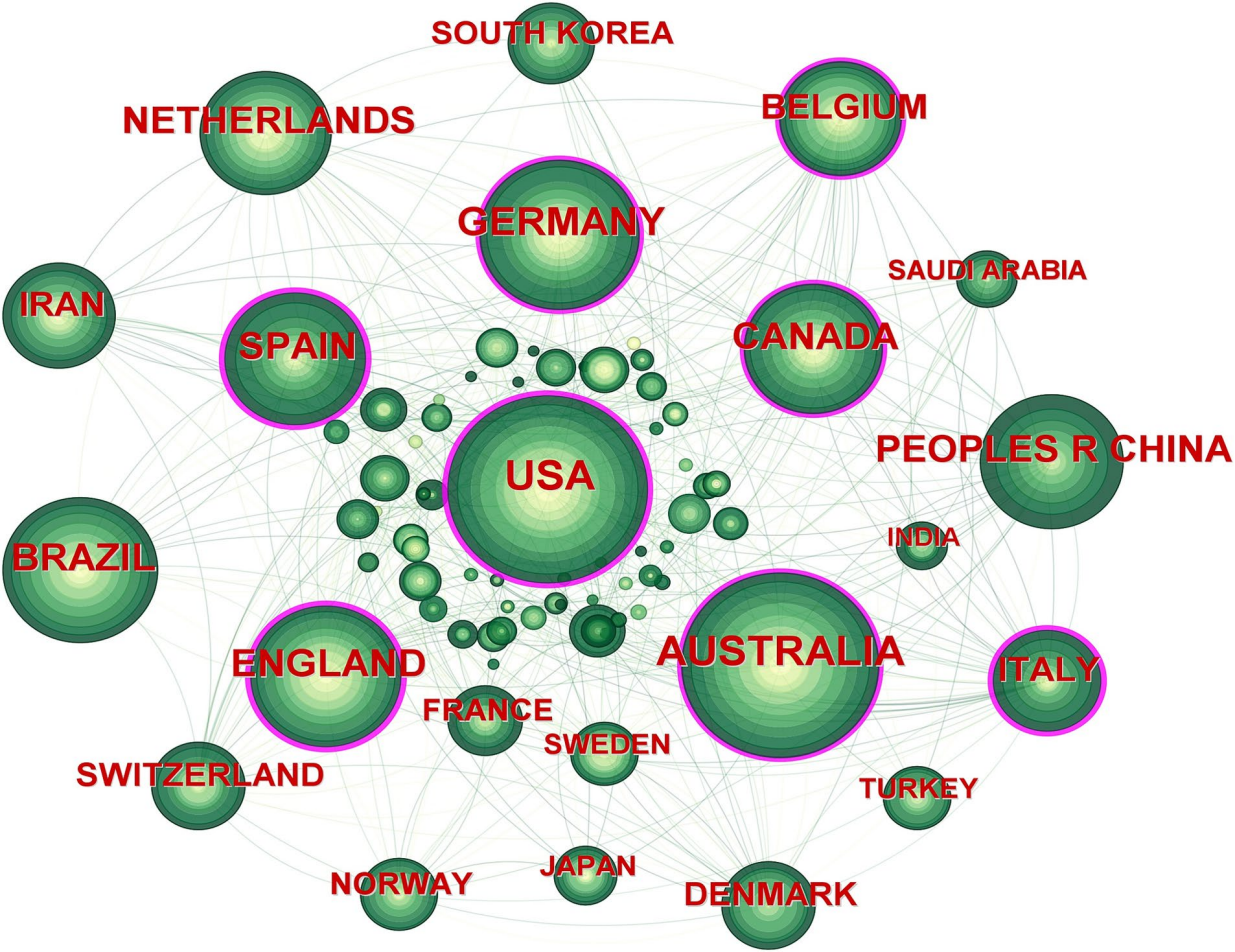
Map of countries researching non-specific low back pain (NSLBP) from 2014 to 2023.

A distribution map of the country partnership network was generated through CiteSpace, consisting of 80 nodes and 496 connectors (Figure 4), representing 2,103 articles from 80 countries. The country with the highest number of published articles was the USA (329 articles, 15.64%), followed by Australia (318 articles, 15.12%), Germany (211 articles, 10.03%) England (193 articles, 9.18%), and Brazil (192 articles, 9.13%). The highest centrality was in the USA (0.29), followed by England (0.24), Spain (0.22), Italy (0.21), and Australia (0.15). The top 10 countries to which the journal publication and centrality belonged are shown in Table 4. In conclusion, the USA has published the most high-quality articles related to NSLBP and has conducted in-depth research on NSLBP, with a good research foundation.

**Table 4:** Top 10 publications and centrality of countries related to non-specific low back pain (NSLBP).

Rank	Publications	Countries	Rank	Centrality	Countries
1	329	USA	1	0.29	USA
2	318	Australia	2	0.24	England
3	211	Germany	3	0.22	Spain
4	193	England	4	0.21	Italy
5	192	Brazil	5	0.15	Australia
6	168	Peoples Republic of China	6	0.15	Belgium
7	163	Spain	7	0.12	Canada
8	163	Canada	8	0.11	Germany
9	161	Netherlands	9	0.08	Netherlands
10	118	Belgium	10	0.08	France

**Figure 5:**
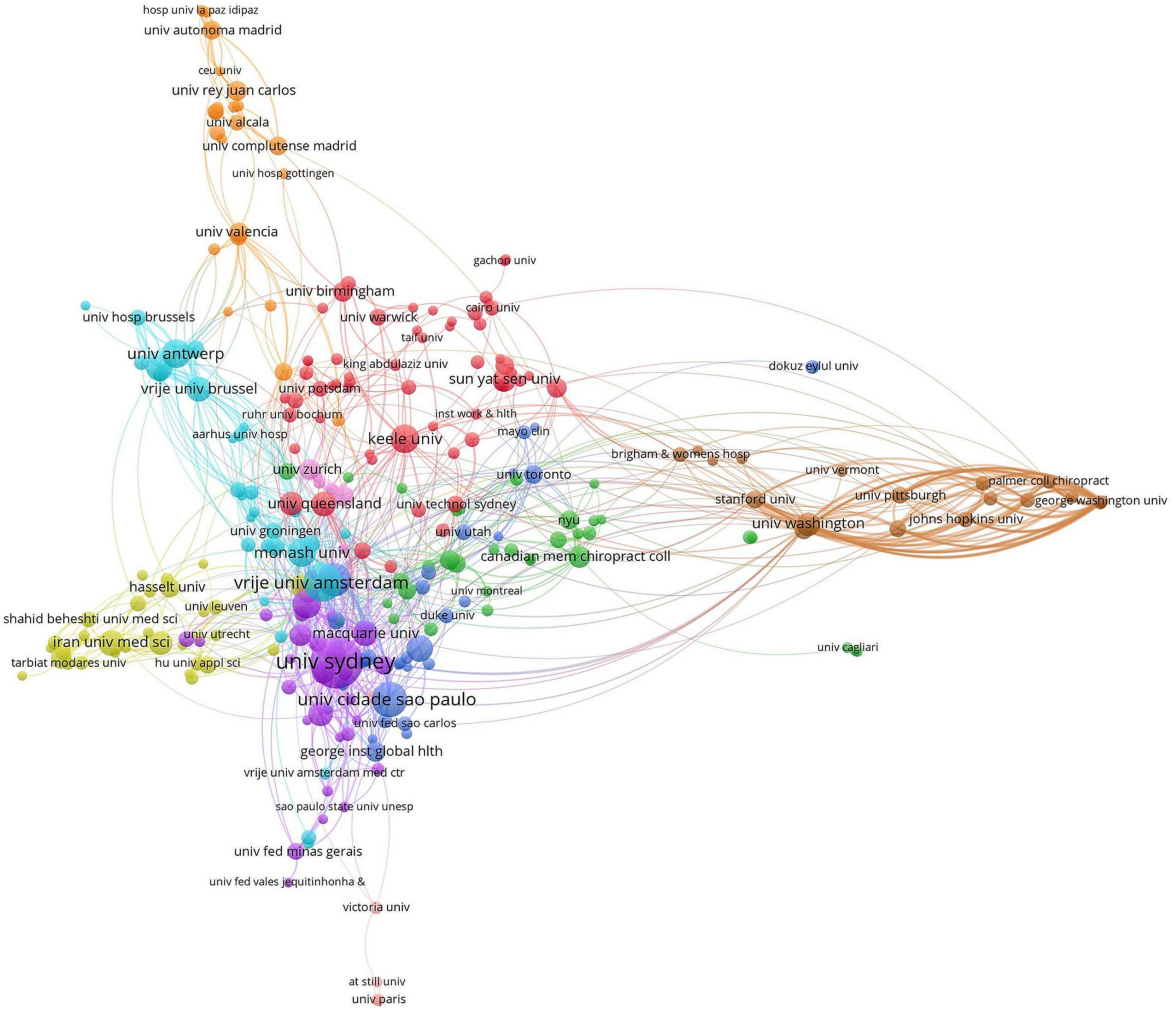
Map of institutions researching non-specific low back pain (NSLBP) from 2014 to 2023.

Research institutions are important places for the production of scientific knowledge, and the distribution of power in the field of research can be understood by analyzing the collaborative relationships among different research institutions. In total, 2,103 articles were published by 367 research institutions dedicated to the study of NSLBP (Figure 5). The institution with the highest number of published articles was the University of Sydney (139 articles, 6.61%), followed by Vrije University Amsterdam (85 articles, 4.04%), Universidade Cidade de São Paulo (72 articles, 3.42%), the University of Southern Denmark (52 articles, 2.48%), and the George Institute for Global Health (45 articles, 2.14%). The highest centrality was found in Vrije Universiteit Amsterdam (0.20), followed by Harvard University (0.16), the University of Sydney (0.11), the University of London (0.11), and Keele University (0.10). The top 10 institutions in terms of article publication and centrality are shown in Table 5. The analysis shows that institutions in countries such as Australia, the USA, the United Kingdom, the Netherlands, and Brazil dominate the field of NSLBP research. Research institutions are mainly concentrated in comprehensive universities, with a small proportion of non-university or specialized research institutions. The various research institutions have close internal connections and relatively weak cross-regional cooperation, lacking cooperation and communication with research institutions from other countries. Implications as well as recommendations for enhancing such collaborations, would provide practical insights and broaden the horizons for researchers.

**Table 5:** Top 10 publications and centrality of institutions related to non-specific low back pain (NSLBP).

Rank	Publications	Institutions	Rank	Centrality	Institutions
1	139	University of Sydney	1	0.20	Vrije Universiteit Amsterdam
2	85	Vrije University Amsterdam	2	0.16	Harvard University
3	72	Universidade Cidade de São Paulo	3	0.11	University of Sydney
4	52	University of Southern Denmark	4	0.11	University of London
5	45	George Institute for Global Health	5	0.10	Keele University
6	43	Curtin University	6	0.09	Monash University
7	38	Keele University	7	0.08	Vrije Universiteit Brussel
8	36	Monash University	8	0.08	University of Queensland
9	35	Vrije Universiteit Brussel	9	0.07	Curtin University
10	35	Ghent University	10	0.06	University of Southern Denmark

### 3.4 Analysis of authors and cited authors

**Figure 6:**
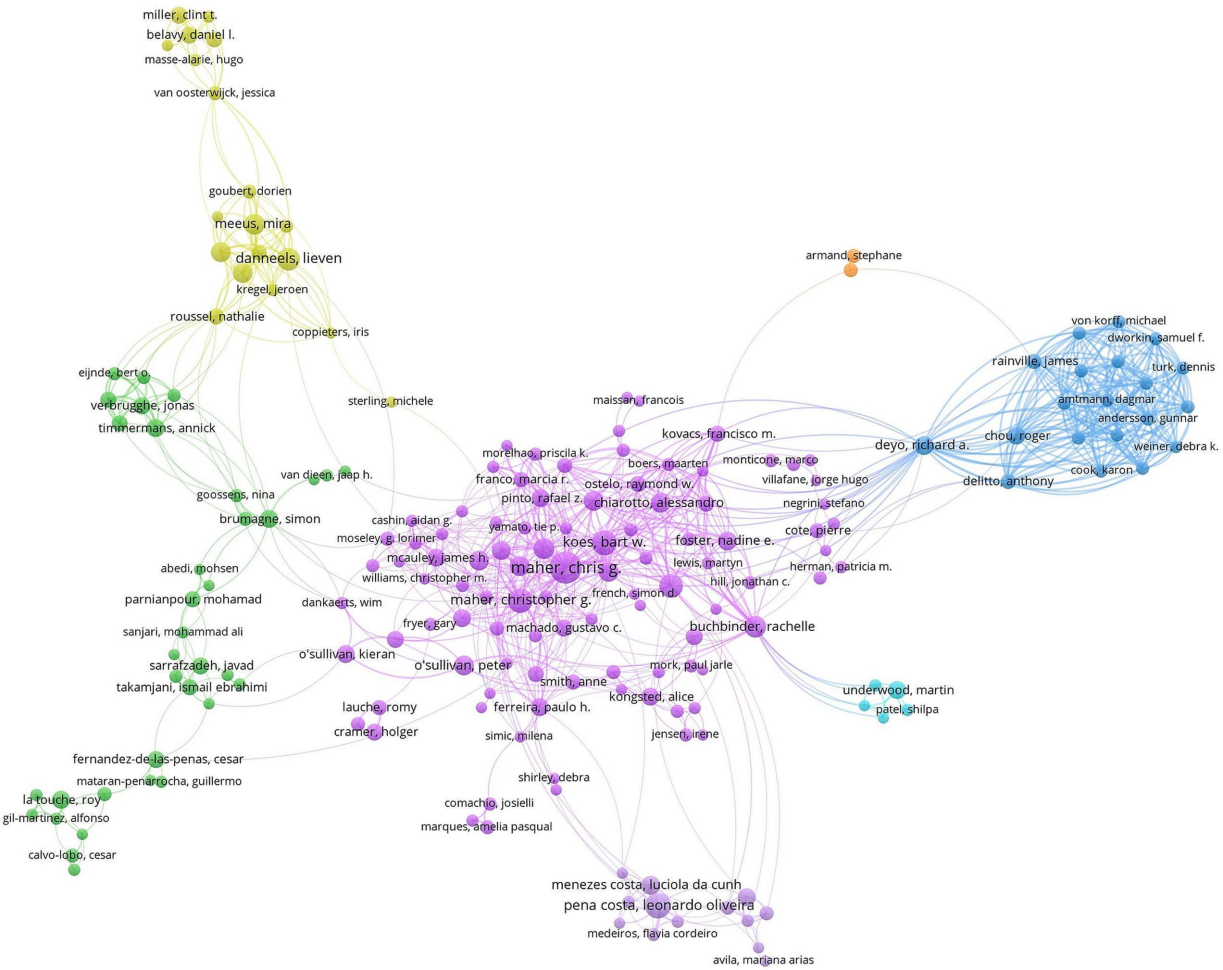
Map of authors related to non-specific low back pain (NSLBP) from 2014 to 2023.

A network map of author collaborations can be used to understand the number of articles published by the author in a certain field and to grasp the degree of cooperation between the research team to which the author belongs (Figure 6). The most prolific author in the study on NSLBP was Maher, Chris G (36 articles), followed by Pena Costa Leonardo Oliveira (23 articles), Danneels, Lieven (19 articles), Maher, Christopher G (17 articles), and Koes, Bart W (17 articles). The top 10 are shown in Table 6. Maher, Chris G is a staff member at the Institute of Musculoskeletal Health in Sydney, dedicated to researching clinical practice guidelines, Healthcare costs in emergency department, opioid use, and systematic reviews of NSLBP. He has provided reliable evidence and made the most prominent contribution to the field of NSLBP research. A comprehensive analysis showed that most of the research teams conducted the research independently, with close internal cooperation and less inter-team cooperation, which may be related to the different specialities and research directions of each team. If the research teams can strengthen cross-team, inter-disciplinary, and inter-specialty cooperation, there will be greater breakthroughs in the research related to NSLBP.

**Table 6:** Top 10 prolific authors related to non-specific low back pain (NSLBP).

Rank	Publications	Author	Rank	Publications	Author
1	36	Maher, Chris G	6	14	Cagnie, Barbara
2	23	Pena costa, Leonardo Oliveira	7	14	Oliveira, Crystian B
3	19	Danneels, Lieven	8	13	Buchbinder, Rachelle
4	17	Maher, Christopher G	9	12	Meeus, Mira
5	17	Koes, Bart W	10	12	Chiarotto, Alessandro

**Figure 7:**
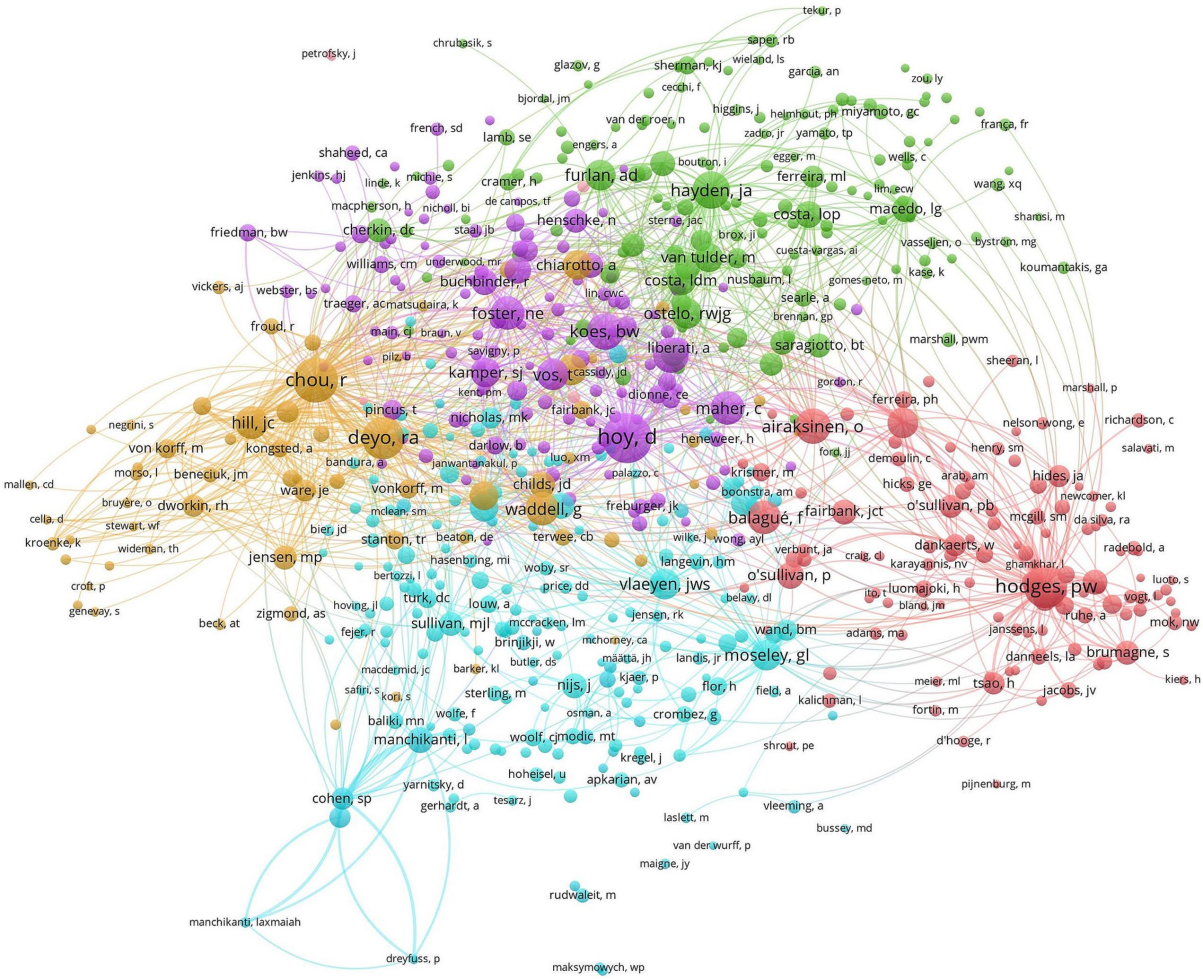
Map of cited authors related to non-specific low back pain (NSLBP) from 2014 to 2023.

The co-citation network map of authors can identify the core authors in a certain research field and show the cross-citation relationship between literature related to NSLBP (Figure 7). The most frequently cited author was Hoy, D (571 articles, 27.15%), followed by Chou, R (468 articles, 22.25%), Hodges, PW (427 articles, 20.30%), Deyo, RA (409 articles, 19.45%), and Hayden, JA (327 articles, 15.55%). The authors with the highest centrality were Baliki, MN (0.07), followed by Hodges, PW, Moseley, GL, George, SZ, and Sullivan, MJL (0.04). Data on the frequency and centrality of the top 10 cited authors are shown in Table 7. In summary, Hoy, D and Baliki, MN have high activity and have conducted extensive research on NSLBP, making positive contributions to the development of this field.

**Table 7:** Top 10 frequency and centrality of cited authors related to non-specific low back pain (NSLBP).

Rank	Frequency	Author	Rank	Centrality	Author
1	571	Hoy, D	1	0.07	Baliki, MN
2	468	Chou, R	2	0.04	Hodges, PW
3	427	Hodges, PW	3	0.04	Moseley, GL
4	409	Deyo, RA	4	0.04	George, SZ
5	327	Hayden, JA	5	0.04	Sullivan, MJL
6	310	Koes, BW	6	0.04	Macedo, LG
7	288	Airaksinen, O	7	0.04	Ferreira, ML
8	287	Hartvigsen, J	8	0.04	Kongsted, A
9	263	Waddell, G	9	0.04	Barked, KL
10	254	Foster, NE	10	0.03	Roland, M

### 3.5 Analysis of cited references

**Figure 8:**
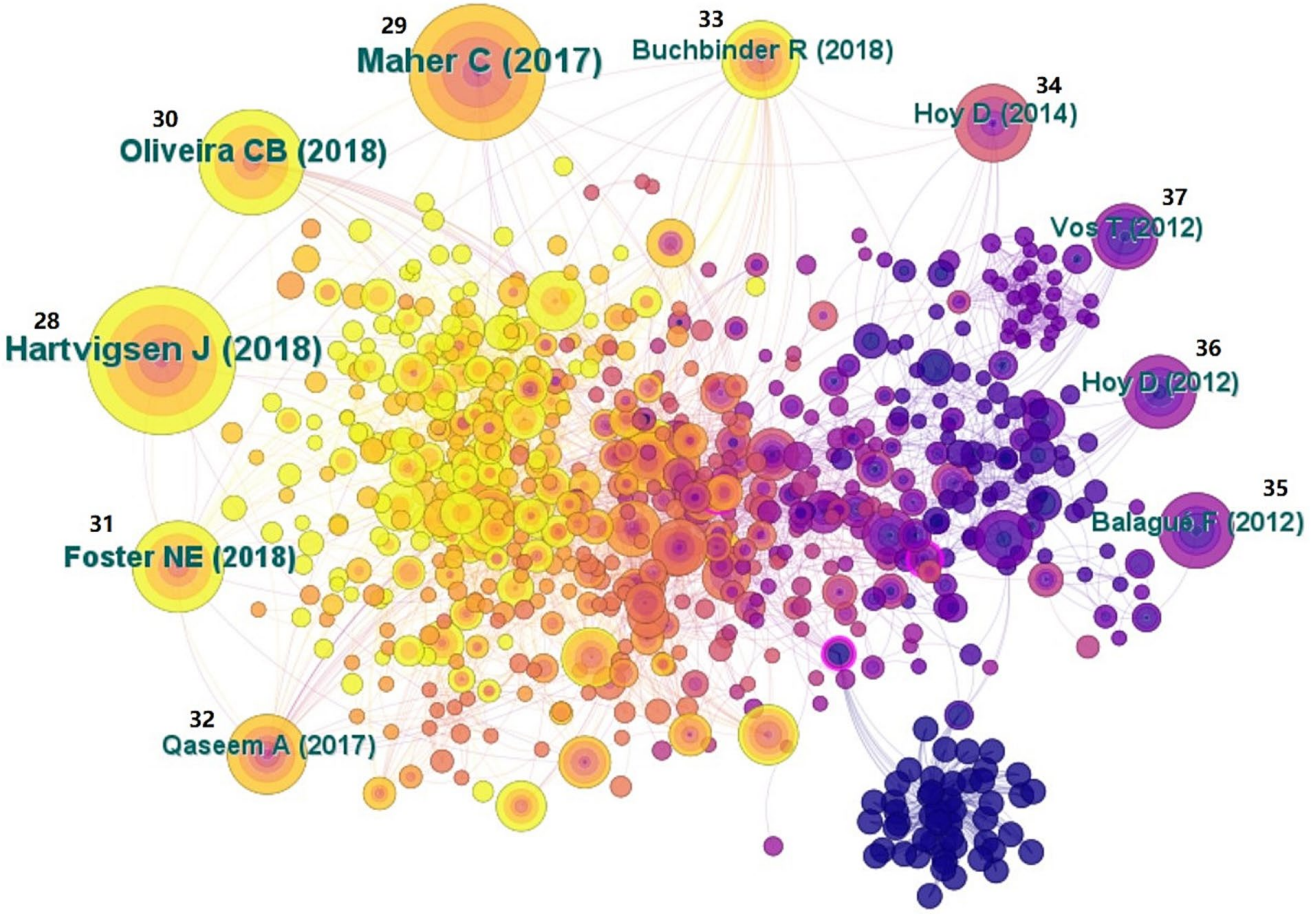
Map of cited references related to non-specific low back pain (NSLBP) from 2014 to 2023.

Analyzing reference co-occurrences is not only beneficial for searching out high-quality literature but also for understanding hot topics related to NSLBP [Figure 8, references (28–37)]. By counting the frequency of references, it is possible to determine the quality of the literature and the degree of repercussions in the professional field. The top 10 frequency rankings of references are shown in Table 8, which shows that these studies are the most highly influential in the field of NSLBP. Centrality measures the likelihood of any shortest path passing through a node in a network, which can guide us in finding the most valuable node in the network. The top 10 references with high centrality are listed in Table 9, references (38–44), indicating that these references play a crucial role in the field of NSLBP.

**Table 8:** Top 10 frequency of cited references related to non-specific low back pain (NSLBP).

Rank	Frequency	References	Author and publication year
1	243	LANCET, V391, P2356. DOI 10.1016/S0140-6736(18)30480-X (28)	Hartvigsen J, 2018
2	209	LANCET, V389, P736. DOI 10.1016/S0140-6736(16)30970-9 (29)	Maher C, 2017
3	133	EUR SPINE J, V27, P2791. DOI 10.1007/s00586-018-5673-2 (30)	Oliveira CB, 2018
4	102	LANCET, V391, P2368. DOI 10.1016/S0140-6736(18)30489-6 (31)	Foster NE, 2018
5	80	ANN INTERN MED, V166, P514. DOI 10.7326/M16-2367 (32)	Qaseem A, 2017
6	74	LANCET, V391, P2384. DOI 10.1016/S0140-6736(18)30488-4 (33)	Buchbinder R, 2018
7	71	ANN RHEUM DIS, V73, P968. DOI 10.1136/annrheumdis-2013-204428 (34)	Hoy D, 2014
8	67	LANCET, V379, P482. DOI 10.1016/S0140-6736(11)60610-7 (35)	Balagué F, 2012
9	63	ARTHRITIS RHEUM-US, V64, P2028. DOI 10.1002/art.34347 (36)	Hoy D, 2012
10	50	LANCET, V380, P2163. DOI 10.1016/S0140-6736(12)61729-2 (37)	Vos T, 2012

**Table 9:** Top 10 centrality of cited references related to non-specific low back pain (NSLBP).

Rank	Centrality	References	Author and publication year
1	0.18	CLIN J PAIN, V29, P907. DOI 10.1097/AJP.0b013e31827a6dd8 (38)	Bunzli S, 2013
2	0.17	MED J AUSTRALIA, V208, P272. DOI 10.5694/mja17.01152 (39)	Almeida M, 2018
3	0.11	EUR SPINE J, V27, P2791. DOI 10.1007/s00586-018-5673-2 (30)	Oliveira CB, 2018
4	0.08	PAIN, V159, P481. DOI 10.1097/j.pain.0000000000001117 (40)	Chiarotto A, 2018
5	0.07	EUR J PAIN, V17, P916. DOI 10.1002/j.1532-2149.2012.00252.x (41)	Fersum KV, 2013
6	0.07	JAMA INTERN MED, V176, P958. DOI 10.1001/jamainternmed.2016.1251 (42)	Shaheed CA, 2016
7	0.07	CLIN REHABIL, V29, P1155. DOI 10.1177/0269215515570379 (43)	Searle A, 2015
8	0.06	EUR J PAIN, V21, P201. DOI 10.1002/ejp.931 (44)	Wong JJ, 2017
9	0.06	LANCET, V389, P736. DOI 10.1016/S0140-6736(16)30970-9 (29)	Maher C, 2017
10	0.06	ANN INTERN MED, V166, P514. DOI 10.7326/M16-2367 (32)	Qaseem A, 2017

**Figure 9:**
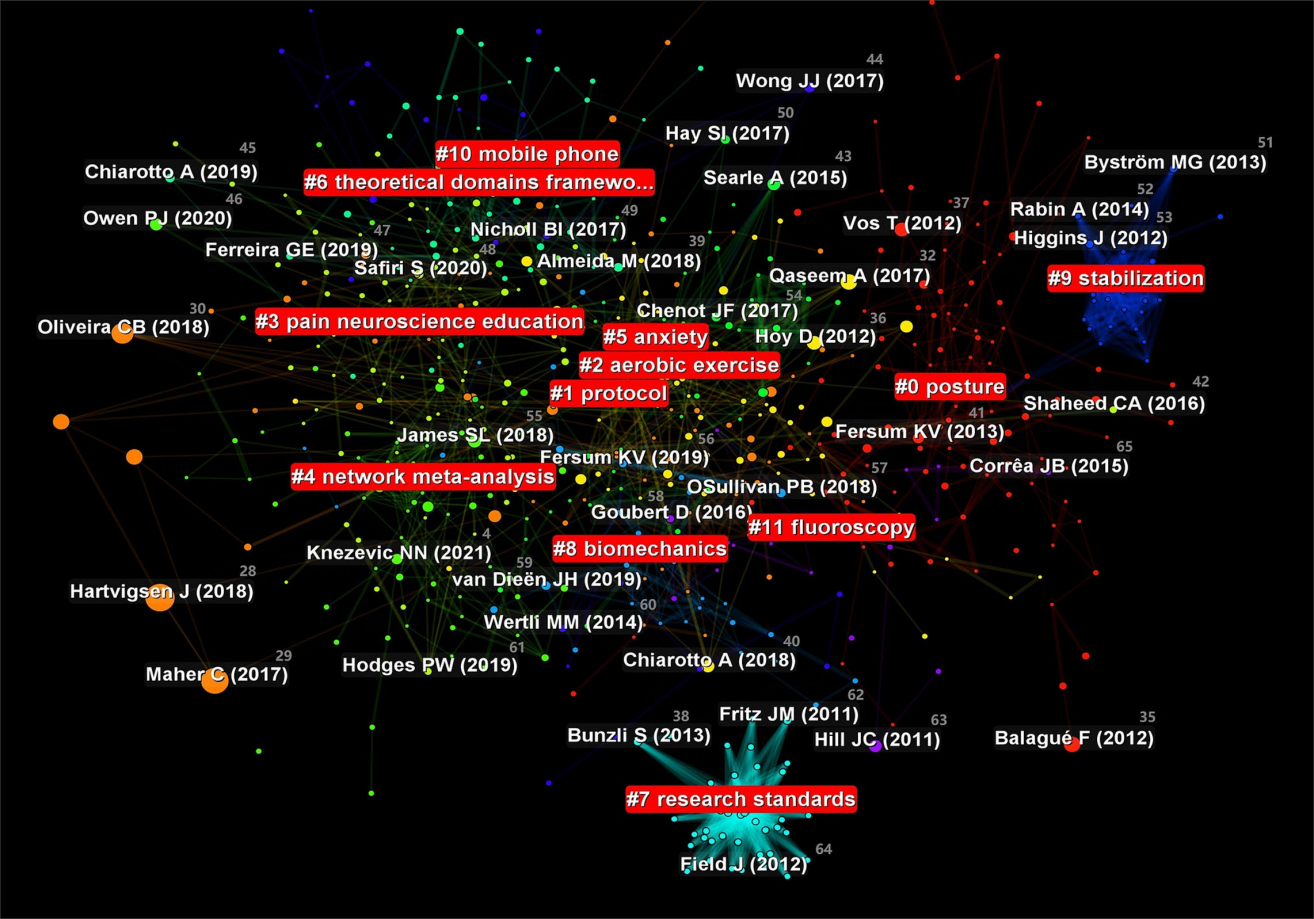
Cluster analysis map of co-citation references related to non-specific low back pain (NSLBP) from 2014 to 2023.

To obtain knowledge on the structure and dynamic change process of a certain research field, we used CiteSpace to automatically extract literature based on the co-occurrence of references. Cluster labels were generated based on the common relationships of the cited literature, and the likelihood ratio algorithm was used for label clustering. In this study, a total of 11 clusters were formed, among which “biomechanics,” “anxiety,” and “pain neuroscience education” were three important clustering results. The clustering module value was *Q* = 0.7588 and the average contour value was *S* = 0.8154 [Figure 9, references (45–65)]. Cluster module value refers to a method of demonstrating the importance and development level of a topic or cluster through specific measurement indicators such as density and centrality. Average contour value is a statistical indicator used to evaluate the quality of clustering algorithm results. It is mainly used to measure the tightness of members within a cluster and the degree of separation between clusters. Generally speaking, *Q* > 0.3 indicates a significantly better clustering structure, while *S* > 0.7 indicates a higher clustering feasibility. Based on the above analysis, research related to NSLBP has high credibility.

The lumbar spine is an important structure for maintaining spinal stability and range of motion, with complex biomechanical characteristics. Biomechanical changes are closely related to the occurrence of NSLBP (66).

There is a bidirectional relationship between NSLBP and anxiety, which often influence each other. The discomfort symptoms caused by NSLBP can lead to negative emotions, such as anxiety, in patients, which in turn accelerates the progression of NSLBP. The clinical application of anti-anxiety drugs to treat NSLBP not only effectively reduces anxiety, but also improves the factors of disability induced by NSLBP (67).

Pain neuroscience education (PNE) is an effective method for treating NSLBP. A single-blind randomized clinical trial showed that PNE achieved better results in reducing motor phobia and altering the perception of LBP intensity compared to purely therapeutic exercise (68).

### 3.6 Analysis of keywords

**Figure 10:**
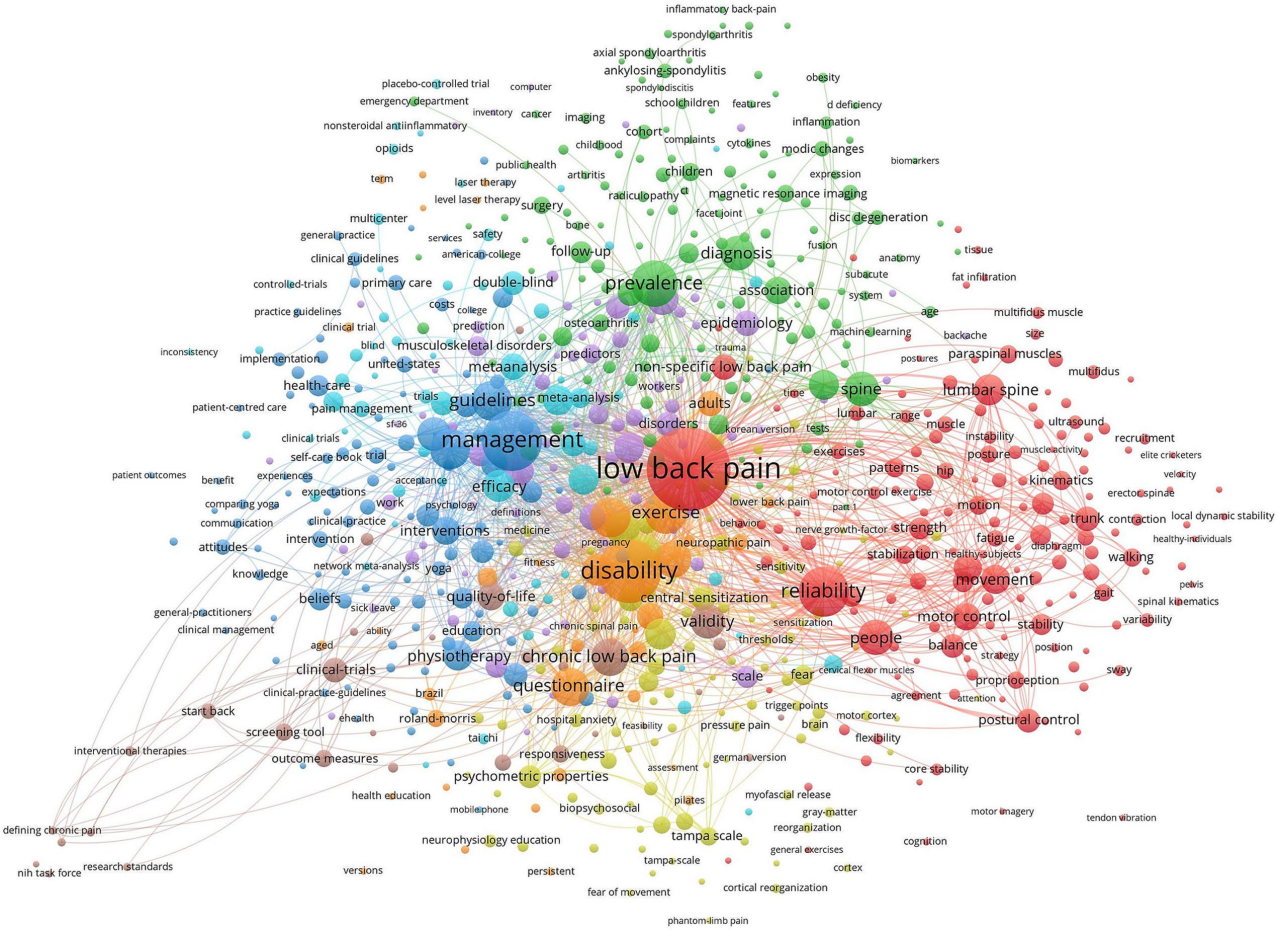
Map of keywords occurrence related to non-specific low back pain (NSLBP) from 2014 to 2023.

**Figure 11:**
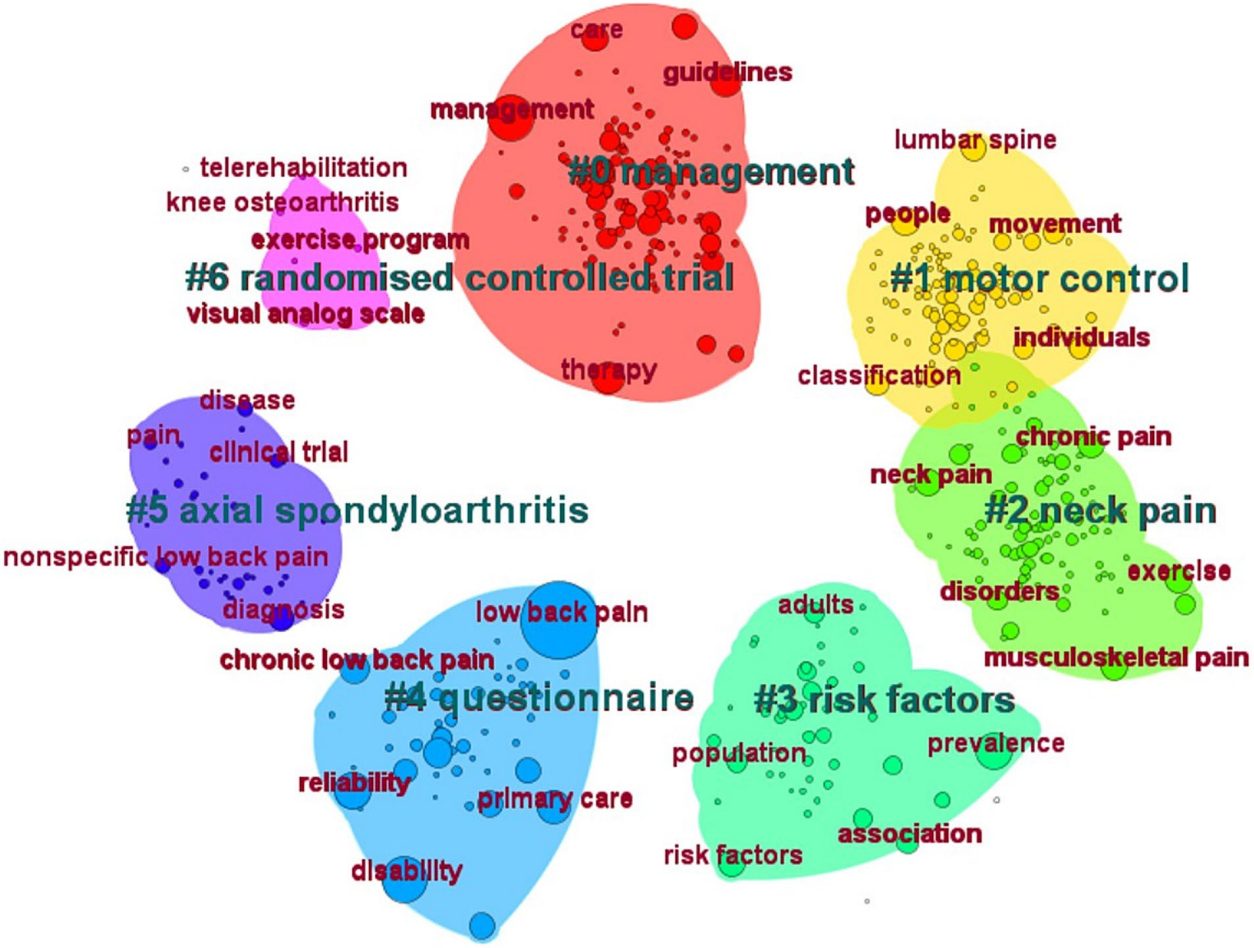
Cluster analysis map of co-citation keywords related to non-specific low back pain (NSLBP) from 2014 to 2023.

Keywords are highly summarized and condensed to the topic of the article. Through the analysis of keyword co-occurrence, we could obtain the central ideas and research hotspots in the field of NSLBP (Figure 10). We found that “low back pain,” “management,” “disability,” “reliability,” and “prevalence” were the most popular keywords (Table 10). Keyword clustering can summarize the main research clusters that have been developed in a field. In this study, a total of seven clusters were formed, among which “motor control” and “neck pain” were two important clustering results. The obtained keywords were clustered and analyzed by the clustering algorithm: *Q* = 0.3401 > 0.3, which indicates that the mapping clusters are well structured, and *S* = 0.7647 > 0.7, which indicates that the clusters have credibility (Figure 11). In general, the clusters are crisscrossed and closely linked. Research trends and hotspots in this field are focused on the therapeutic methods and associated diseases.

**Table 10:** Top 10 frequency and centrality of keywords related to non-specific low back pain (NSLBP).

Rank	Keyword	Frequency	Rank	Keyword	Centrality
1	Low back pain	1,019	1	Performance	0.05
2	Management	357	2	Activation	0.04
3	Disability	348	3	Central sensitization	0.04
4	Reliability	228	4	Health care	0.04
5	Prevalence	211	5	Outcm	0.04
6	Primary care	185	6	Rehabilitation	0.04
7	Therapy	162	7	Spine	0.04
8	Guidelines	156	8	Association	0.03
9	Randomized controlled trial	145	9	Burden	0.03
10	Chronic low back pain	141	10	Clinical trials	0.03

**Figure 12:**
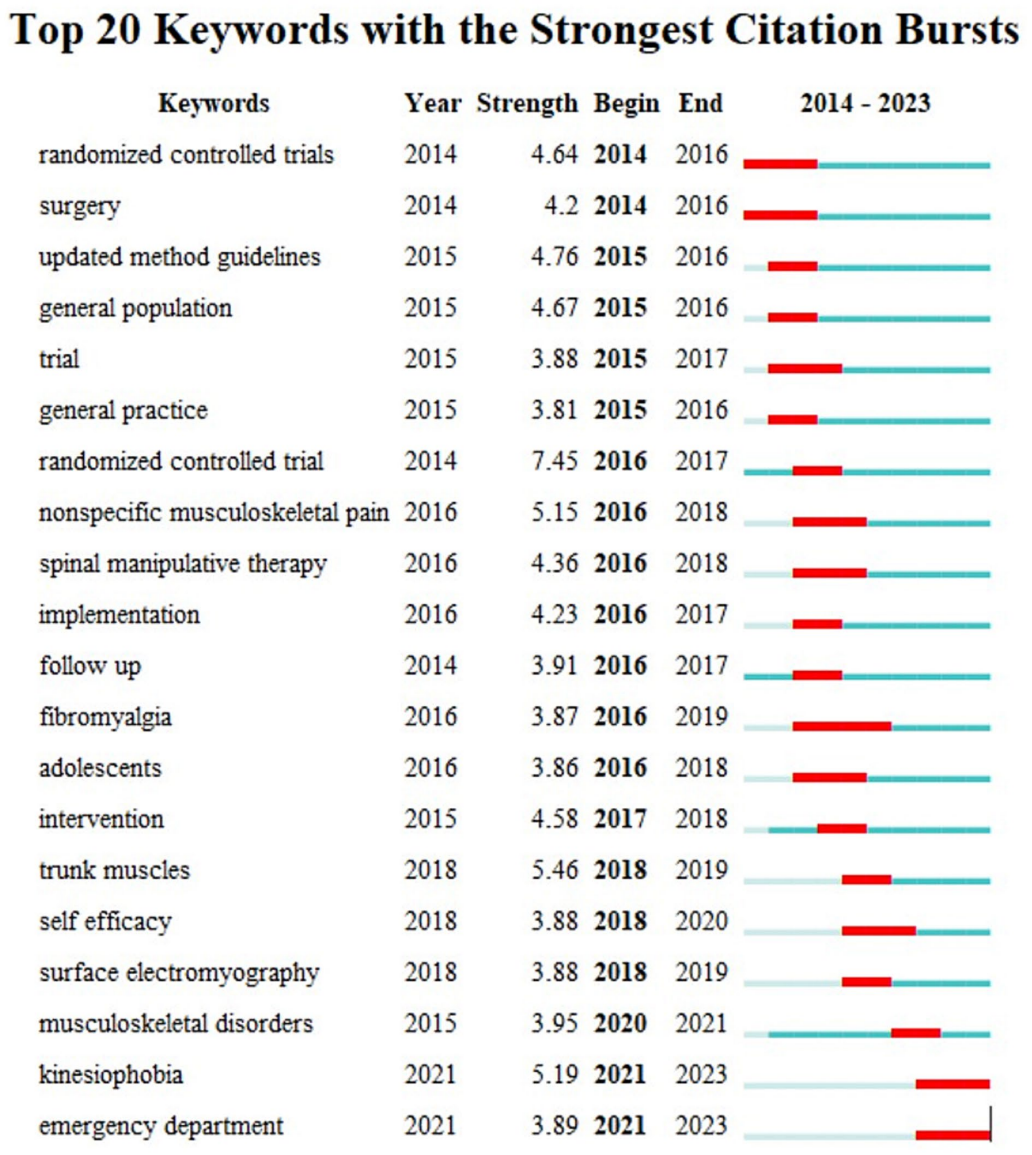
Top 20 keywords with the strongest citation bursts. The bolded **Begin** column demonstrates the start year of the keyword.

**Figure 13:**
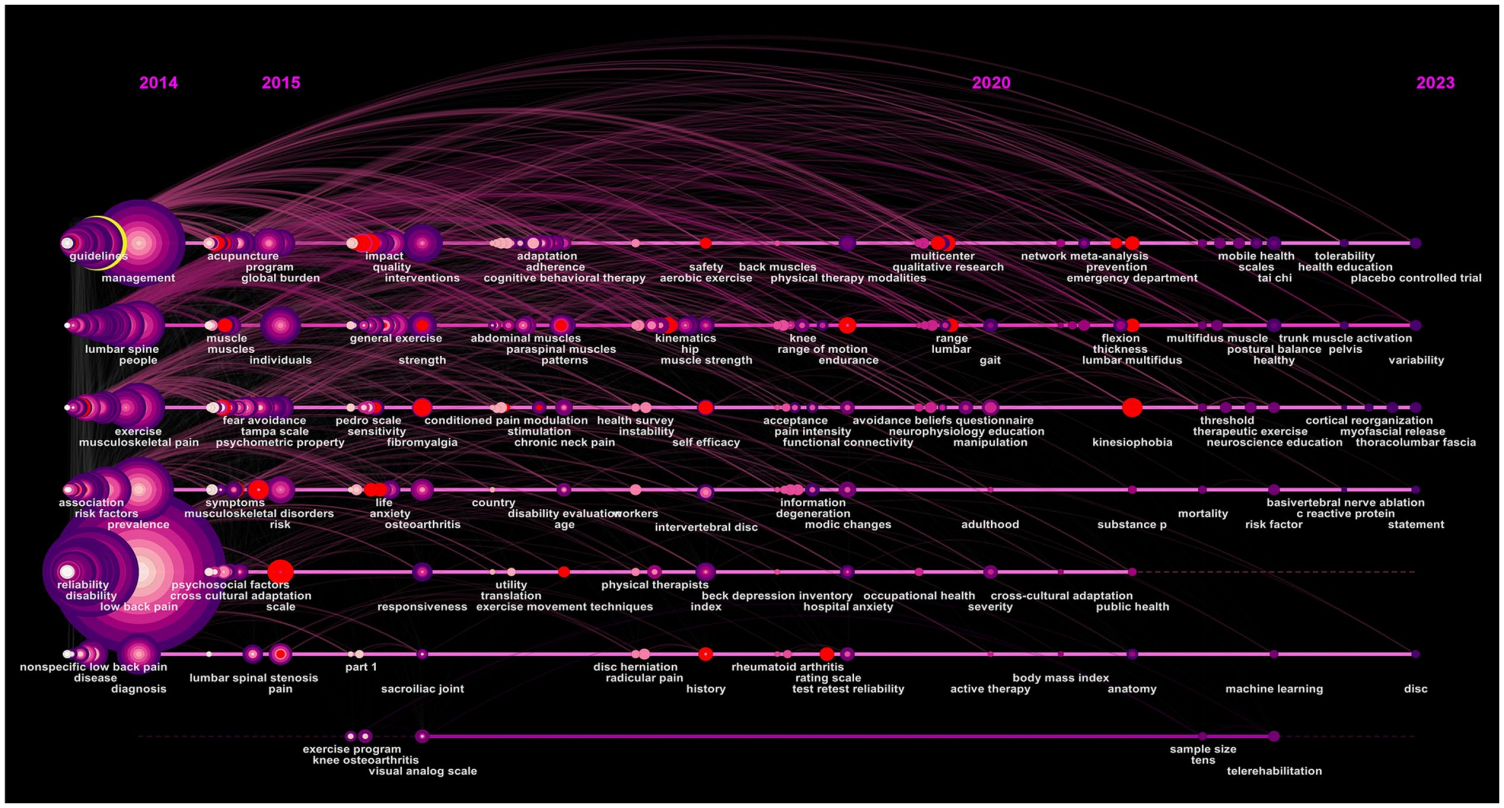
Timeline view of keywords on non-specific low back pain (NSLBP).

Keyword burst is a category of words that appear frequently and grow rapidly in a short period of time. It can roughly determine the research outbreaks and development trends that appear in each period of time and can be used to make a general prediction of the research trend. The top 20 keywords with the strongest citation bursts in 2014–2023 are shown in Figure 12. From 2014 to 2016 experts and scholars focused their research on treatment protocols including the design of randomized controlled trials follow-ups and updating method guidelines. The research direction of 2017–2023 is richer; we should pay special attention to high incidence population (adolescents) treatment methods (spinal manipulative therapy) treatment ideas (trunk muscle) and associated diseases (non-specific musculoskeletal pain fibromyalgia and musculoskeletal disorders) with NSLBP. The keyword timeline chart can reflect the development of the relevant hotspots in the field in each time period and through the analysis of the research vein the main development and evolution trend of the field can be grasped to a certain extent. The figure shows that acupuncture has a long time span (Figure 13) which indicates that in recent years researchers have used acupuncture as an alternative therapy to treat NSLBP. Furthermore it shows that research in this field is deepening and treatment methods are rich and diverse in recent years the proportion of adolescent cases of NSLBP has been continuously increasing. Backpack weight psychological issues lack of physical exercise obesity and abnormal posture are considered important factors causing NSLBP in adolescents (69, 70).

Motor control (MC) is one of the most popular and widely used treatment options for NSLBP, with multiple short-term and long-term advantages in reducing pain and disability (71). It has been suggested that this may be related through modulation of rsFC between the cerebellum and areas involved in sensory-discriminative processing of noxious and somato-sensory stimuli, affection, and cognition (72).

A study observed changes in the levels of inflammatory mediators, including tumor necrosis factor-alpha (TNFα), interleukin (IL)-6, and TNF soluble receptor type 2 (sTNFR2), which ultimately confirmed that spinal manipulative therapy (SMT) has good effects on both acute and chronic NSLBP (73). It could be inferenced that SMT may provide sufficient afferent stimulus to autonomic nervous system and provoke an anti-inflammatory reflex modulating the response of in flammatory cells (73).

As an alternative therapy, acupuncture has attracted the attention of researchers. A systematic review and meta-analysis showed that acupuncture treatment of acute/subacute NSLBP has significant advantages over oral drug treatment, with high safety and significant efficacy (*p* < 0.00001, *I*^2^ = 90%, SMD = −1.42, 95% CI: −2.22, −0.62) (74).

The trunk muscles are an important muscle group involved in lower back activity, playing a crucial role in maintaining lumbar stability. Trunk muscle dysfunction is often considered a key factor in inducing NSLBP (75, 76).

## 4 Conclusion

This study used 2,103 articles on NSLBP obtained from the WOSCC database from 2014 to 2023 as raw materials and used CiteSpace and VOSviewer as information visualization software to draw a series of knowledge graphs. Bibliometrics were used for statistical analysis to visually display the research status of NSLBP in the past decade, and objectively predict future hotspots and frontiers. In the past decade, the annual publication volume of NSLBP has shown an overall upward trend year by year, with obvious temporal stages and great development potential. In total, the 2,103 articles contain six types of literature, with the highest proportion being original research articles (1,633 articles, 77.65%). The literature has been published in 200 journals, with *BMC Musculoskeletal Discourses* (90 articles, 4.28%) having the highest number of publications, and the *British Medical Journal* having the highest IF (105.7). The USA (329 articles, 15.64%) had the highest publication volume, and the University of Sydney (139 articles, 6.61%) was the research institution with the highest production. Furthermore, Maher, Chris G (36 articles, 1.71%) was the author with the most published articles, and Hoy, D (571 articles, 27.15%) was the most frequently cited author. The most cited of articles is “Non-specific low back pain” published in the LANCET, with 1,256 citations.

The hotspots and frontiers of NSLBP can be summarized as follows: In recent years, adolescents have become a high-risk group for NSLBP. PNE, MC, SMT, and acupuncture are effective means to treat NSLBP. Biomechanics and trunk muscles as entry points are effective ideas for the treatment of NSLBP. Furthermore, anxiety, neck pain, non-specific musculoskeletal pain, fibromyalgia, and musculoskeletal disorders are diseases that are closely related to NSLBP. In the future, attention should be paid to the design of research plans, increasing the research intensity of randomized controlled trials, strengthening follow-ups, and providing timely updating of guidelines, thus resulting in higher quality and high-level scientific evidence for NSLBP research.

We found that the distribution of research institutions on NSLBP in the past decade has been uneven, with the vast majority concentrated in comprehensive universities and only a small portion in non-university or specialized research institutions. The research institutions were relatively scattered, with little academic cooperation and communication between them. Most research teams were in the independent research stage, resulting in close cooperation within the team and reduced cooperation between teams. Authoritative research institutions and core teams had not yet been formed. Therefore, in-depth cooperation and academic exchanges among different countries, institutions, teams, and authors from multiple disciplines, perspectives, and methods are strongly recommended to achieve complementary advantages, strengthen research capabilities, broaden research perspectives, and tap into academic resources, thus striving to release higher-level research results (77–80).

Although bibliometric methods provide a quantitative analytical tool, there are still some limitations in practical applications. It mainly focuses on the external characteristics of the literature, such as author, country and institution, without involving in-depth analysis of the content of the literature. This method is suitable for analyzing the macro structure and trends of literature, but it is insufficient for exploring the potential information and deep meanings in literature. In the future, other research methods such as content analysis should be combined to obtain more comprehensive and in-depth research results (21, 81, 82).

## Data Availability

The original contributions presented in the study are included in the article/supplementary material, further inquiries can be directed to the corresponding authors.
